# Factors contributing to a coronavirus disease 2019 (COVID-19) outbreak on a mixed medical-surgical unit in a Canadian acute-care hospital

**DOI:** 10.1017/ash.2022.288

**Published:** 2022-09-08

**Authors:** Megan K. McCallum, Glenn Patriquin, Ian R.C. Davis, Tammy MacDonald, Daniel Gaston, Jason J. LeBlanc, Yahya Shabi, B. Lynn Johnston

**Affiliations:** 1 Infection Prevention and Control, Nova Scotia Health, Halifax, Nova Scotia, Canada; 2 Department of Pathology and Laboratory Medicine, Nova Scotia Health, Halifax, Nova Scotia, Canada; 3 Department of Pathology, Faculty of Medicine, Dalhousie University, Halifax, Nova Scotia, Canada; 4 Department of Medicine, Nova Scotia Health and Department of Medicine, Faculty of Medicine, Dalhousie University, Halifax, Nova Scotia, Canada

## Abstract

**Objective:**

To identify preventable factors that contribute to the cross transmission of severe acute respiratory coronavirus virus 2 (SARS-CoV-2) to patients in healthcare facilities.

**Design:**

A case–control study was conducted among inpatients on a coronavirus disease 2019 (COVID-19) outbreak unit.

**Setting:**

This study was conducted in a medical-surgical unit of a tertiary-care hospital in Nova Scotia in May 2021.

**Patients:**

Patients hospitalized on the unit for at least 12 hours and healthcare workers (HCW) working on the unit within 2 weeks of outbreak declaration were included.

**Methods:**

Risk factors for SARS-CoV-2 infection were analyzed using simple and multiple logistic regression. Whole-genome sequencing (WGS) was performed to identify SARS-CoV-2 strain relatedness. Network analysis was used to describe patient accommodation.

**Results:**

SARS-CoV-2 infections were identified in 21 patients (29.6%) and 11 HCWs (6.6%). WGS data revealed 4 distinct clades of related sequences. Several factors likely contributed to the outbreak, including failure to identify SARS-CoV-2, a largely incomplete or unvaccinated population, and patient wandering behaviors. The most significant risk factor for SARS-CoV-2 infection was room sharing with an infectious patient, which was the only factor that remained statistically significant following multivariate analysis (odds ratio [OR], 9.2l; 95% confidence interval [CI], 2.04–41.67; *P* = .004).

**Conclusions:**

This outbreak likely resulted from admission of 2 patients with COVID-19, with subsequent transmissions to 17 patients and 11 staff. WGS and bioinformatics analyses were critical to identifying previously unrecognized nosocomial transmissions of SARS-CoV-2. This study supports strategies to reduce nosocomial transmissions of SARS-CoV-2, such as single-patient rooms, promotion of COVID-19 vaccination, and infection prevention and control measures including management of wandering behaviors.

Since the beginning of the coronavirus disease 2019 (COVID-19) pandemic,^1^ there have been reports of severe acute respiratory syndrome coronavirus 2 (SARS-CoV-2) transmission to patients and healthcare workers (HCW) within healthcare facilities.^2–21^ Factors contributing to these outbreaks have included patient accommodation in multibed rooms or bays,^6–8,11,12,17^ lack of infection prevention and control precautions due to failure to identify patients admitted with or incubating SARS-CoV-2,^6–10,20^ and nosocomial exposure to a patient with COVID-19.^15–20^ Rhee et al^22^ demonstrated that consistent application of infection prevention and control measures can reduce or prevent the spread of SARS-CoV-2 in healthcare settings. However, outbreaks in healthcare facilities continue. To better understand potentially preventable factors that may contribute to the transmission of SARS-CoV-2 in healthcare facilities, we investigated an outbreak of COVID-19 that occurred on a mixed medical-surgical unit of a tertiary-care hospital in Nova Scotia, Canada, in May 2021. This outbreak occurred during Canada’s third wave of COVID-19, which was driven predominantly by the α (alpha) variant of concern (VOC; lineage B.1.1.7).

## Materials and methods

### Population and study design

The study period was 14 days prior to date of outbreak declaration (day 0) to 28 days after the last patient case, when the outbreak was declared over. The study population included all patients hospitalized on the unit for at least 12 hours between day −14 and day 0, and all HCWs who worked on the unit during that time, as well as during the outbreak. A case was defined as any patient or HCW with laboratory-confirmed SARS-CoV-2 detected from a respiratory tract specimen by a nucleic acid amplification test (NAAT). Both patients and HCWs were included in the microbiological analysis and outbreak curve. A case–control study design was used to examine patient variables and outcomes. The source of the infection (community or healthcare associated) was defined using the Canadian Nosocomial Infection Surveillance Program (CNISP) case definition (Appendix 1).^23,24^ We used the CNISP criteria for attributing the cause of death to a viral respiratory infection (Appendix 1).^23,24^ A fully vaccinated person was defined as having had a dose of a single-dose vaccine or the second dose of a 2-dose vaccine at least 14 days prior to a positive test (case) or admission to the outbreak unit (noncase). Partially vaccinated was defined as those with 1 dose of a 2-dose vaccine at least 14 days prior to a positive test (case) or admission to the outbreak unit (noncase). Those with no vaccine doses or with 1 dose of any vaccine <14 days prior to a positive test (case) or admission to the outbreak unit (noncase) were considered unvaccinated.

This study was determined to be a quality improvement initiative that did not require patient consent.

### Data collection and analysis

Patient data were collected from patient charts, visitor logs, bed census reports, discussion with the nurse manager regarding patient activities, the provincial COVID-19 vaccination record, and laboratory databases. Patient data included the following: age, sex, admission and discharge dates, room and bed number, admission diagnosis, comorbidities, COVID-19 vaccination status, dates of all SARS-CoV-2 tests, date of first positive SARS-CoV-2 test, SARS-CoV-2 infection status of roommate(s), outcome at 30 days after SARS-CoV-2 infection for cases and at discharge or day 28 for noncases if still in hospital. Throughout the 14 days preceding the outbreak, the following data were recorded: patient bed movements and mobilization within and off the unit, visitors, COVID-19 daily screening results, assigned shift nurse, and receipt of an aerosol-generating medical procedure (AGMP). Data on HCW cases, as well as the total number of staff who worked on the unit within the specified duration above were provided by the occupational health, safety, and wellness department.

Descriptive statistics were used to summarize patient characteristics and risk factors. Univariate analysis using simple logistic regression was used to identify risks for infection and outcome. *P* ≤ .05 was considered statistically significant. Multiple logistic regression was conducted including variables identified as statistically significant in the univariate analysis. Analyses were conducted using Microsoft Excel 365 software (Microsoft, Redmond, WA) and SPSS Statistics version 28 software (IBM, Armonk, NY). A network analysis was conducted using Pajek version 5.3 software to identify patients who were roommates of a case during the outbreak period.

### Whole-genome sequencing (WGS)

Nasopharyngeal (NP) swabs underwent validated commercial NAAT testing for SARS-CoV-2, as previously described.^25^ WGS was performed at the National Microbiology Laboratory (NML) using the Oxford Nanopore MinION sequencing instrument. Consensus sequences were generated using the nCoV-tools analysis pipeline, based on Next-Strain/Augur (https://nextstrain.org/ and https://github.com/jts/ncov-tools). Incomplete sequences (<50% coverage of the whole genome) were not included in the analysis (ie, patients 3 and 17, and staff B). Partial genomes (>50% but <95%) (ie, for patients 6 and 16, and staff C) were included in the default analysis.

Consensus sequences from all WGS of SARS-CoV-2 isolates submitted from Nova Scotia between March 30, 2021, and May 25, 2021, were also included in this analysis. Sample whole-genome consensus sequences were realigned, and a new phylogenetic tree was estimated using the augur pipeline with default settings. Multiple sequence alignments were created using MAFFT software^26^ and the maximum-likelihood phylogenetic tree was constructed using IQ-TREEz software.^27^ The phylogenetic tree was visualized using Auspice software (auspice.us). A strict analysis, with all partial genomes removed, was also performed. It did not change the overall shape of the phylogenetic tree or clusters in which outbreak-related sequences fell.

## Results

**Fig. 1.**
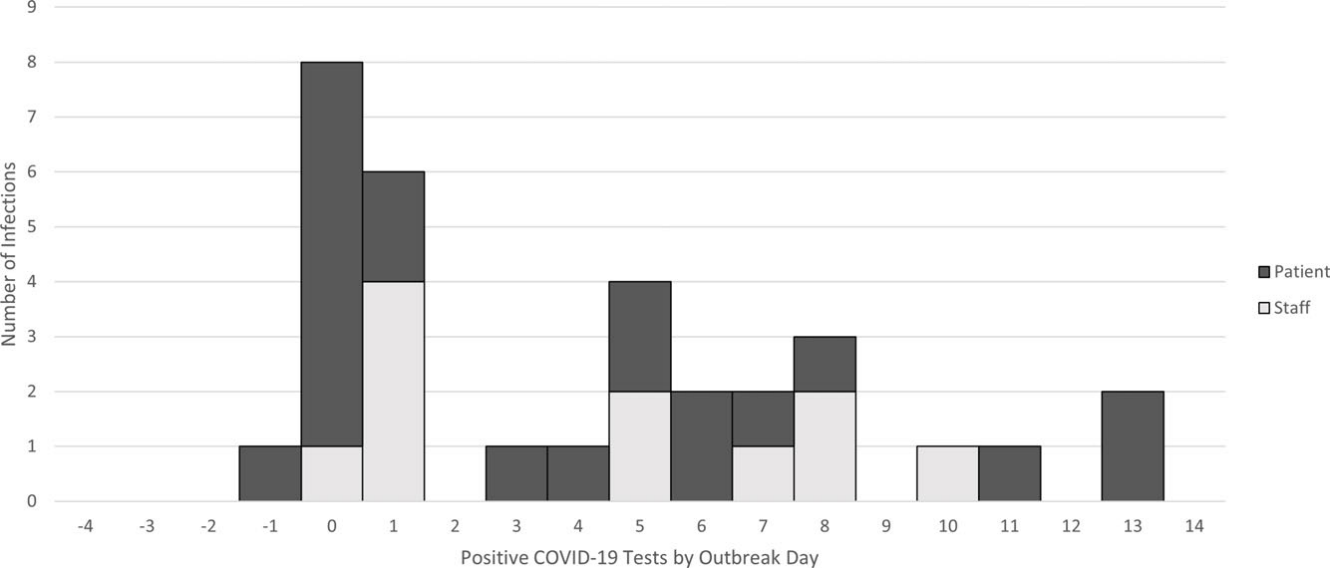
Epidemic curve of COVID-19 infections in patients and staff by day of first positive test in relation to outbreak onset.

Of 71 patients hospitalized on the outbreak unit from day −14 until day 0, 21 (29.6%) were diagnosed with SARS-CoV-2 infection between day −1 and day 13 (Fig. 1). Of the 30 patients discharged from the outbreak unit before day −4, none were subsequently diagnosed with COVID-19. Of 167 staff, 11 (6.6%) tested positive. A review of staffing assignments was unable to identify any potential staff-to-patient transmissions.

Table 1 summarizes the results of the univariate and multivariate analyses. The median age of COVID-19 patient cases and noncases was similar, with no difference in the proportion who were male or female. According to the CNISP definition, 17 patient cases were defined as acquired in the healthcare facility and 4 as community associated. Patients who shared a room with a patient with COVID-19 during their infectious period were at very high risk of subsequently developing COVID-19 (odds ratio [OR], 15.33; 95% confidence interval [CI], 4.02–58.46; *P* < .001). Other risk factors for COVID-19 identified on univariate analysis were being a roommate of a patient with dementia, frequent wandering on the unit, and being a roommate of a patient who frequently wandered on the unit. On multivariate analysis, sharing a room with a patient with COVID-19 during their infectious period was the only factor that remained statistically significant (OR, 9.2; 95% CI, 2.04–41.67; *P* = .004).

**Table 1.** Characteristics and Exposures Among Patient Cases and Non-Cases

Variable	Total (n=71), No. (%)^a^	Cases (n=21), No. (%)^a^	Non-cases (n=50), No. (%)^a^	Odds Ratio (CI) Univariate Analysis	Odds Ratio (CI) Multivariate Analysis
Age, median y (range)	74 (26–103)	76 (31–92)	73 (26–103)	1.01 (0.98–1.03) *P* = .682	
**Sex**					
Male	29 (41)	10 (48)	19 (38)	0.67 (0.24–1.89) *P* =.453	
Female	42 (59)	11 (52)	31 (62)		
Roommate of patient with COVID-19	46 (65)	19 (90)	27 (54)	8.09 (1.70–38.49) *P* =.009	2.09 (0.32–13.81) *P* = .446
Roommate of patient with COVID-19 during their infectious period	16 (23)	12 (57)	4 (8)	15.33 (4.02–58.45) *P* < .001	9.21 (2.04–41.67) *P* = .004
Wandering patient	9 (13)	6 (29)	3 (6)	6.27 (1.39–28.17) *P* = .017	4.24 (0.67–26.98) *P* = .127
Roommate of wandering patient	31 (44)	14 (67)	17 (34)	3.88 (1.32–11.43) *P* = .014	1.02 (0.23–4.52) *P* = .982
Dementia	14 (20)	7 (33)	7 (14)	3.07 (0.92–10.29) *P* = .069	
Delirium	12 (17)	4 (19)	8 (16)	1.24 (0.33–4.65) *P* = .755	
Roommate of dementia patient	33 (46)	15 (71)	18 (36)	4.44 (1.47–13.47) *P* = .008	2.39 (0.60–9.55) *P* = .217
**Vaccination status**					
Fully	4 (6)	2 (10)	2 (4)	3.70 (0.46–29.64) *P* = .218	
Partially	18 (25)	8 (38)	10 (20)	2.96 (0.93–9.47) *P* = .067	
Not vaccinated (ref)	47 (66)	10 (48)	37 (74)		
Unknown	2 (3)	1 (5)	1 (2)	3.70 (0.21–64.51) *P* = .370	
**Outcome**					
Deceased	11 (15)	6 (29)	5 (10)	3.60 (0.96–13.52) *P* = .058	
Alive	60 (85)	15 (71)	45 (90)		

Note. CI, confidence interval; ref, reference.

a Units unless otherwise specified.

Most patients (66%) had not received any doses of COVID-19 vaccine prior to the outbreak, with no significant difference between cases and noncases. Only 6 patients (8%) had been exclusively in a single room, none of whom developed COVID-19. Being the roommate of a patient undergoing AGMPs was not a statistically significant risk factor; however, this was likely due to the small number of patients on AGMPs and a lack of statistical power and should be examined in future studies. Moreover, 27 patients experienced 32 within-unit transfers from day −14 onward; however, this was not a statistically significant risk factor for acquiring COVID-19.

Furthermore, 11 patient deaths (15%) occurred during the outbreak: 6 among the 21 patients with COVID-19 (mortality rate, 29%), and 5 among the 50 patients who did not have COVID-19 (mortality rate, 10%). The difference in mortality between patients with and without COVID-19 approached statistical significance on univariate analysis. In 3 patients, COVID-19 was considered the cause of death, and in 2 patients, death was considered unrelated to COVID-19. In the remaining patient, COVID-19 was considered a contributing factor.

**Fig. 2.**
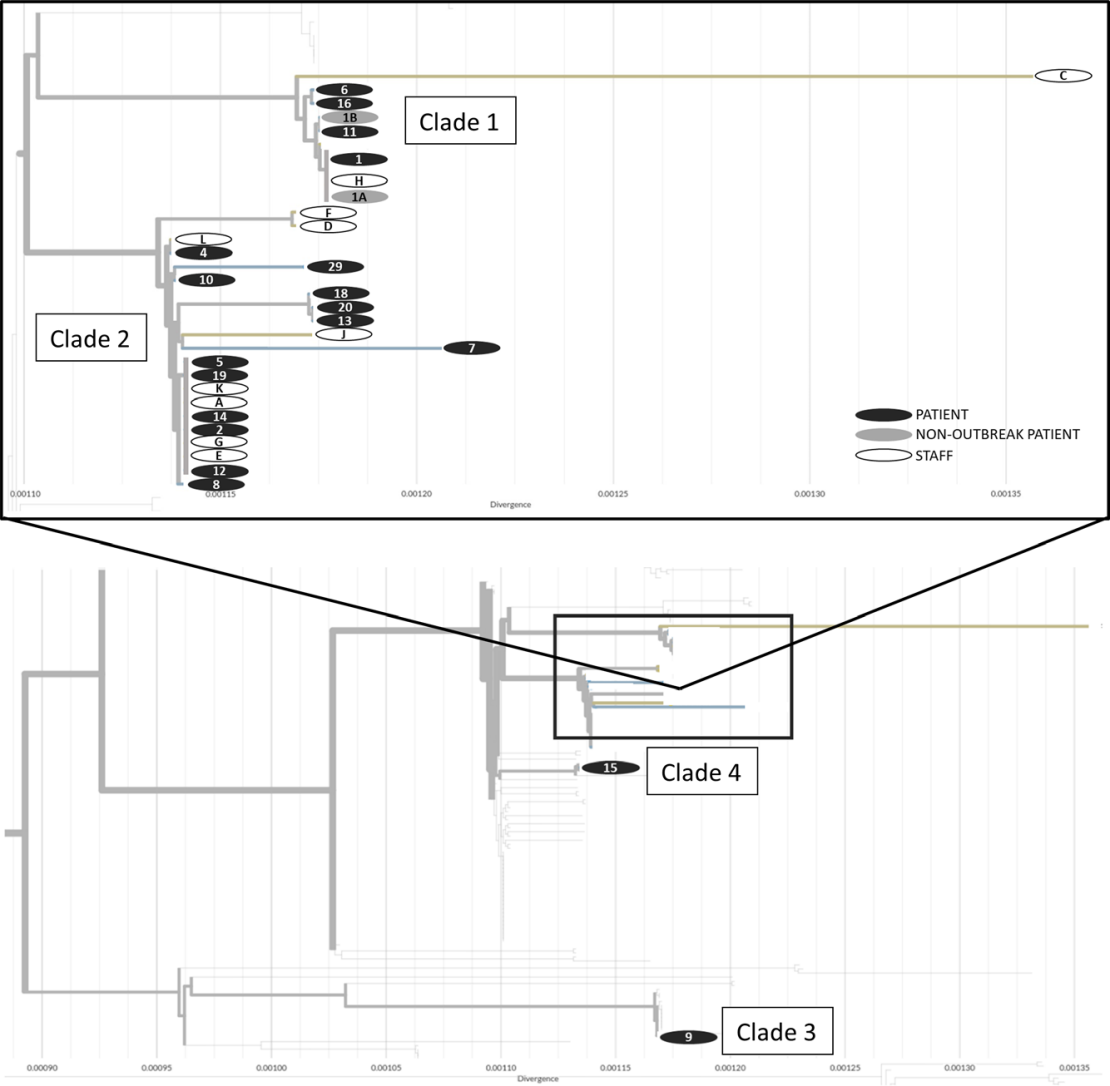
Phylogenetic tree showing genetic distance between all available SARS-CoV-2 specimens collected in Nova Scotia from March 30, 2021, to May 25, 2021.

All available sequences from positive SARS-CoV-2 samples from patient and HCW cases were of the α VOC (PANGO lineage B.1.1.7); however, nucleotide polymorphisms within these lineages allowed phylogenetic analysis and characterization of genome sequences into 4 distinct clades of related sequences (Fig. 2). Clades 3 and 4 consisted of a single case each, and the other 2 clades (1 and 2) included 17 of the remaining 19 patient cases and 10 of 11 staff. The 2 cases (patients 9 and 15) with unique clades better matched community sequences unrelated to the outbreak. Among the 2 larger clades, 1 included 4 patients and 2 HCWs (clade 1) and 1 included 13 patients and 8 HCWs (clade 2). Clade 1 included 3 individuals (1A and 1B) who were not among the outbreak cases, but each had a community epidemiological link to 1 of the outbreak cases. The 2 large distinct clades contain 2 community-associated cases (patients 1 and 7) and the related transmissions in the unit. A review of the charts of these 2 patients revealed that the clinical course was in keeping with community-acquired acquisition. Both had been admitted with respiratory symptoms but had initial NP swabs that were negative for SARS-CoV-2 and were taken off precautions.

**Fig. 3.**
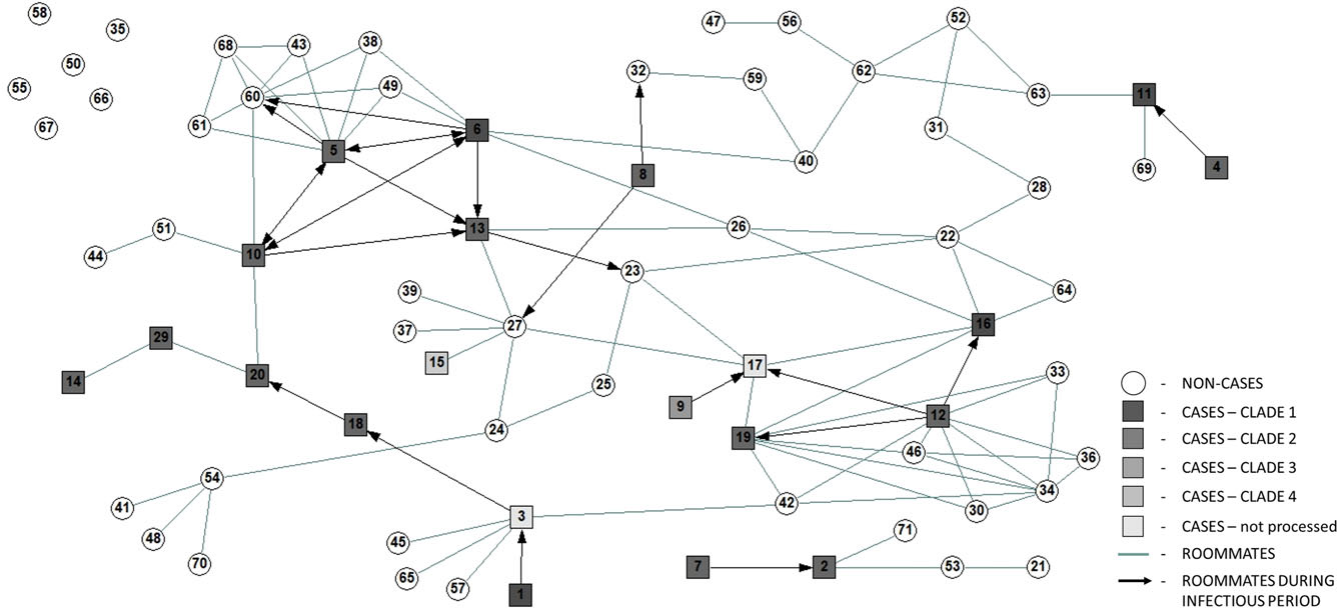
Cluster map depicting room sharing relationships among patients on the outbreak unit in the 14 days prior to and 7 days after declaring the outbreak.

Figure 3 displays the results of the patient network analysis according to clades identified through WGS for SARS-CoV-2 positive patients. Also, 6 patients did not share a room with any other patient during the outbreak, and a cluster of 5 patients did not connect to the larger group of patients.

## Discussion

This COVID-19 outbreak has similarities to other hospital-associated outbreaks.^6–10,20^ Factors that contributed to the outbreak included failure to identify SARS-CoV-2 in admitted patients during the initial stages of infection, a largely incomplete or unvaccinated population, and patient behavioral factors such as a lack of compliance with physical distancing and mask wearing, with some patients wandering on the hospital unit. Roommates of COVID-19 patients were shown to be at a significantly higher risk of acquiring SARS-CoV-2. Finally, our findings highlight the benefits of using WGS during outbreak investigations, not only to confirm transmission events but also to identify previously unrecognized transmission events.

The outbreak likely resulted from the admission of 2 patients on outbreak day −4 in whom the diagnosis of COVID-19 was incorrectly dismissed, due to false-negative SARS-CoV-2 tests, with subsequent transmission to 17 additional patients and 11 staff. The 30% patient attack rate was somewhat higher than reported in other studies^4,10,13,14^ but lower than that reported by Karan et al.^14^ Of the 30 patients discharged from the unit prior to outbreak day −4, none had a prior or subsequent COVID-19 diagnoses, which further supports the hypothesis that the index patients were admitted to the unit on outbreak day −4. A third patient (clade 3) was admitted with acute COVID-19, but that admission to the unit was brief (<24 hours) and did not result in any transmissions. For each of these 3 patients, the diagnosis of COVID-19 was incorrectly excluded shortly after their admission based on at least 1 negative test for SARS-CoV-2 and additional precautions were not continued upon admission to the unit. This finding highlights the importance of considering the potential for false-negative diagnostic tests and maintaining precautions until the patient’s symptoms have improved and/or an alternative diagnosis has been made to avoid the risk of a sizeable hospital-acquired outbreak.

In this study, we have shown the benefits of the outbreak investigation of adding WGS analysis. Other reports have highlighted the utility of WGS in outbreak investigation,^3,5,9,12,13,16,18,20,21^ including its ability to identify complex outbreaks characterized by introduction of infections by >1 source or refute the original transmission hypothesis. WGS supported this study’s hypothesis of the introduction of SARS-CoV-2 onto the unit by 2 patients who presented to the hospital with respiratory symptoms, one of whom was later found to have had community contact with COVID-19. The community source of the other patient’s SARS-CoV-2 infection was never identified. WGS confirmed the hypothesis that the single cases associated with the 2 unique clades were unrelated to the outbreak, with 1 of these cases suspected to have acquired the infection in the community from a relative with COVID-19. The other patient, while meeting the CNISP definition for healthcare-associated infection, fit more with community acquisition as the patient’s earlier hospitalization had been brief (2 days), with no infected roommates at any point.

Transmission between roommates was supported by the risk-factor analysis showing that sharing a room with a patient with COVID-19 during their infectious period was the most significant risk factor for SARS-CoV-2 infection. Although wandering behaviors in either cases or noncases did not reach statistical significance on multivariate analysis, they did reach statistical significance on univariate analysis. Patients who had a wandering roommate or a roommate with dementia were more likely to acquire COVID-19 than patients who did not have a roommate with one of these factors. Patients who wandered were more likely to acquire COVID-19 than nonwanderers. Two other studies have anecdotally identified the potential risk posed by wandering patients,^12,28^ which is supported by the findings of this study. We did not detect a statistically significant protective effect of being exclusively in a single room prior to the outbreak; however, only 6 patients met this criterion, none of whom developed COVID-19. In 4 patients with COVID-19, a likely source of acquisition could not be ascertained through examination of staff assignments and bed or roommate details. Other outbreaks have reported that the source of infection cannot always be determined.^6,11^ Given the wandering behavior of some patients and generally nonexistent mask use and/or physical distancing among patients when in common areas, it is entirely conceivable that exposures took place between patients who were not roommates. This hypothesis was supported by combining the network analysis (Fig. 3) and WGS alignment, which demonstrated that some transmission events were not explained fully by roommate relationships. These observations reaffirm the importance of single rooms in minimizing the risk of cross transmission of infection in healthcare settings and the difficulties in supervising the movement of patients with delirium and dementia in hospitals, with the subsequent potential for nosocomial spread. This finding highlights the roles that physical distancing and mask wearing play in prevention in all settings, not just the community.

We were unable to conclusively identify the direction of transmission between all infected patients and HCWs beyond the 2 index cases being the most likely source of the outbreak. HCWs who acquired COVID-19 early in the outbreak (positive test dates of outbreak day 0 and +1) were likely exposed by patients because no COVID-19 staff infections occurred prior to outbreak day −4. After the outbreak was recognized, when both HCWs and patients had tested positive, it was not possible to identify whether transmissions among HCWs were entirely patient to HCW or whether some HCW-to-HCW transmission occurred. Based on WGS, positive test dates, staff work schedules and patient assignments, we were unable to identify a situation in which a patient was likely to have acquired COVID-19 from an HCW.

In this small cohort, vaccination did not appear to confer a protective effect against acquiring COVID-19, but most patients had not received any vaccine prior to the outbreak. This finding demonstrates that patients admitted to hospital may be among the most vulnerable populations and unable to easily access COVID-19 vaccines. At the very least, healthcare facilities should take the opportunity to identify unvaccinated patients early in their admission and provide the vaccine to them.

A strength of this study was the ability to account for the outcomes of all the patients, the access to all COVID-19 testing on the cohort before, during, and after the outbreak. Another strength was the availability of WGS on the patients and HCWs, as well as individuals who were not part of the outbreak. However, the study had several limitations. The variables related to patient movement on the unit were determined from chart review and nurse manager recollection, which is likely to be incomplete. Bias is less likely a factor because not all the patients had developed COVID-19 when the charts were reviewed, and the nurse manager conducted the interviews. Other limitations include a lack of information about HCWs who did not test positive for SARS-CoV-2. Although it was possible to determine shift schedules for all HCWs who tested positive for SARS-CoV-2 and room assignments for nursing staff who tested positive for SARS-CoV-2, all HCW movements and interactions during these shifts could not be accounted for. A small number of patients were involved in this outbreak, which produced insufficient power to definitively identify all risk factors.

In summary, the findings of this study demonstrate that SARS-CoV-2 WGS and bioinformatics analysis were critical tools needed to confirm that 2 unique unrecognized COVID-19 cases resulted in nosocomial transmission to patients and HCWs. Shared patient rooms resulting in exposure to an infected patient as a roommate, patient behaviors characterized by not maintaining physical distancing and/or reliable mask wearing, failure to identify patients admitted with COVID-19, and a largely unvaccinated patient population undoubtedly contributed to this outbreak. Facility design that favors single rooms for patients, promotion of COVID-19 vaccination and public health measures for inpatients, and strategies to manage wandering behaviors in patients are likely to contribute to preventing the transmission of SARS-CoV-2 in healthcare settings.

## Supplementary material

For supplementary material accompanying this paper visit https://doi.org/10.1017/ash.2022.288. click here to view supplementary material
