# Supplementary material for: Factors contributing to a coronavirus disease 2019 (COVID-19) outbreak on a mixed medical-surgical unit in a Canadian acute-care hospital
Source: Antimicrob Steward Healthc Epidemiol. 2022 Sep 8;2(1):e151. doi: 10.1017/ash.2022.288 (PMC9726552; doi:10.1017/ash.2022.288)
Supplement: Supplementary file 1 [file S2732494X22002881sup001.pdf]

*Appendix 1. CNISP COVID-19 Case Definitions and Attributable Mortality*[24]

| **COVID-19 Case Definitions** | |
|---|---|
| Healthcare-associated acquired in your acute care facility (HA-YAF) | Symptom onset ≥ 7[1] calendar days after admission to the reporting hospital and using best clinical judgement (e.g. symptom onset < 7 days but known epi link to a positive case)<br><br>OR<br><br>If the patient is readmitted with a positive test < 7 days after discharge from hospital and using best clinical judgement. |

[1] 7 days was chosen as the cut-off to attribute acquisition to the hospital based on a current estimate of a median incubation of 4 days (IQR 2-7 days) for hospitalized patients with COVID-19. This is a conservative estimate and can be later re-coded based on the date of admission, symptom onset and test date if required. https://www.cdc.gov/coronavirus/2019-ncov/hcp/clinical-guidance-management-patients.html

| | |
|---|---|
| Healthcare-associated any other healthcare exposure (HA-Other) | Any patient who is identified with COVID-19 not acquired at your facility that is thought to be associated with any other healthcare exposure (e.g. another acute-care facility, long-term care, or rehabilitation facility etc.). |
| Community-associated: | No exposure to healthcare that would have resulted in this infection (using best clinical judgement) and does not meet the criteria for a healthcare-associated infection. |
| **Attributable Mortality** | |
| VRI was the cause of death | The patient had no other condition that would have caused death during this hospitalization |
| VRI contributed to the death | VRI exacerbated an existing condition that led to the patient's death |
